# Development of a Stereovision-Based Technique to Measure the Spread Patterns of Granular Fertilizer Spreaders

**DOI:** 10.3390/s17061396

**Published:** 2017-06-15

**Authors:** Simon R. Cool, Jan G. Pieters, Dejan Seatovic, Koen C. Mertens, David Nuyttens, Tim C. Van De Gucht, Jürgen Vangeyte

**Affiliations:** 1 Department of Biosystems Engineering, Ghent University, Coupure Links 653, 9000 Gent, Belgium; Jan.Pieters@ugent.be; 2 Fisheries and Food Research, Institute of Agricultural, Burg. Van Gansberghelaan 115, 9820 Merelbeke, Belgium; Koen.Mertens@ilvo.vlaanderen.be (K.C.M.); David.Nuyttens@ilvo.vlaanderen.be (D.N.); Tim.Vandegucht@ilvo.vlaanderen.be (T.C.V.D.G.); Jurgen.Vangeyte@ilvo.vlaanderen.be (J.V.); 3 Faculty of Information Management & Media, University of Applied Sciences, Moltkestrasse 30, 76133 Karlsruhe, Germany; Dejan.Seatovic@hs-karlsruhe.de

**Keywords:** fertilizer, spread pattern, stereovision

## Abstract

Centrifugal fertilizer spreaders are by far the most commonly used granular fertilizer spreader type in Europe. Their spread pattern however is error-prone, potentially leading to an undesired distribution of particles in the field and losses out of the field, which is often caused by poor calibration of the spreader for the specific fertilizer used. Due to the large environmental impact of fertilizer use, it is important to optimize the spreading process and minimize these errors. Spreader calibrations can be performed by using collection trays to determine the (field) spread pattern, but this is very time-consuming and expensive for the farmer and hence not common practice. Therefore, we developed an innovative multi-camera system to predict the spread pattern in a fast and accurate way, independent of the spreader configuration. Using high-speed stereovision, ejection parameters of particles leaving the spreader vanes were determined relative to a coordinate system associated with the spreader. The landing positions and subsequent spread patterns were determined using a ballistic model incorporating the effect of tractor motion and wind. Experiments were conducted with a commercial spreader and showed a high repeatability. The results were transformed to one spatial dimension to enable comparison with transverse spread patterns determined in the field and showed similar results.

## 1. Introduction

The applied quantity of N-fertilizer has a direct effect on agricultural productivity, environmental pollution, food security, ecosystem health, human health and economic prosperity [1]. A recent study [2] illustrates the large environmental impact of fertilizer in a wheat-to-bread supply chain. The use of ammonium nitrate fertilizer alone accounted for 43.4% of the overall Global Warming Potential (GWP) and 34.1% of the Eutrophication Potential (EP) in the production of a loaf of bread. According to [1] the most immediate solutions to this fertilizer problem reside in increasing Nitrogen Use Efficiency (NUE) while maintaining high yield, through improved crop plant physiology but also through improved agronomic practices such as precision agriculture. Granular fertilizer is mostly spread by centrifugal fertilizer spreaders [3,4] due to their robustness, simplicity, low cost and large working widths [5,6]. The spreading process however is very sensitive to errors [7,8]. Poor fertilizer quality, erroneous configuration of the spreader or the lack of correct setting tables can lead to deviations between the desired and the actual spread pattern of fertilizer in the field [9,10]. Spreaders are often used without being calibrated for the specific fertilizer applied [6] which can result in local under- and over-application of fertilizer [9]. Spreader evaluation and calibration at farm level is traditionally done by using an in-field setup of collection trays, removing collected material and weighing the contents [11] which is a laborious and time consuming process [12]. In case of precision agriculture, close loop control systems are required with appropriate sensors to manage the local fertilization rate [7,13]. Different techniques have been investigated in literature to monitor parameters of fertilizer particles leaving the spreading vanes and/or use these parameters to determine the spread pattern using a ’predict rather than collect’ approach. Generally, these approaches use a ballistic flight model to determine the landing positions of individual particles. To calculate the trajectories, input data is necessary to initiate the equations of motion. This data can be simulated (modeling approach) or measured (hybrid approach). Different authors [14,15,16,17,18,19,20,21,22] determined sets of differential equations with analytical or numerical solutions to describe motion of an individual particle along the spreading vanes. They illustrate that the particle dynamics depends on fertilizer physical properties and spreader parameters and provide some insight in the spreading process, however they ignore particle interactions. The Discrete Element Method (DEM) model by [23,24] takes these interactions into account. The model shows promising results for short vanes and a reduced disk speed (300 rpm). However, increasing deviations were found at higher (more realistic) disc speeds [25,26]. Furthermore, quantitative simulations require specific physical characteristics of fertilizer particles that are difficult to measure. Because particle motion on the disk proves difficult to predict, hybrid approaches have been developed, combining the use of a ballistic model with the measurement of parameters of particles leaving the vanes. In [27] three dimensional (3D) particle velocities in a 20 mm × 20 mm area were measured using a technique based on the ultrasonic Doppler frequency shift using one transmitter and three receivers. The technique was also used to determine particle size however this proved less accurate. An optical sensor was developed in [28] using two photosensitive arrays, a light source and a converging and diverging lens to determine radial velocity and diameter of particles passing through a 30 mm orifice. Particle velocity and diameter were estimated with 5% and 2% accuracy respectively. In the work reported in [29] the sensor was fitted to a rotating arm with an encoder to scan the area around the spreading disk. However, the mounting configuration implied that only the radial velocity component could be measured, which according to the authors was the reason for the under-estimation of the spreading width. In [13], asingle-camera stroboscopic image acquisition technique and corresponding image processing algorithms were presented to measure horizontal outlet angle and velocity of particles. An imaging technique was developed by [30] to derive the same parameters based on motion blurred images, on which particle trajectories appeared as straight lines due to continuous lighting during camera exposure. Due to the fact that only two-dimensional information was obtained, both single camera techniques were applicable only in the case of flat spreading disks (nowadays rarely used in practice due to a limited working width), assuming that particle trajectories near the spreader were parallel to the image plane. However even in the latter case, particles leave the spreader with a vertical angle [5], causing errors in the calculated diameters and velocity vectors. The motion blurring technique was extended in the study of [7] to determine 3D velocity components of particles based on spreader configuration parameters and the measured horizontal outlet angle. The system depends on a kinematic relationship between the particle velocity on the disk and the spreader configuration (disk parameters) and horizontal outlet angle [21] thereby ignoring particle interactions. Because of the blurring technique, particle size could not be determined [31] developed a test bench (CEMIB) with a row of collection trays with weighing sensors, capable of measuring the spread pattern in two spatial dimensions in a test hall (40 m × 10 m). A stereo-configuration of high speed cameras and corresponding image processing algorithms was used by [32] to determine particle positions and velocities (based on two subsequent positions) in three dimensions, independent of the spreader configuration. However, due to the large field of view (1 m^2^), which resulted in a small particle resolution, and the limited distance between cameras, the uncertainty of the predicted 3D positions (and subsequently the velocities) was too high. Furthermore, illumination intensity and variability were found insufficient and the orientation of the camera with respect to the spreader needed to be determined manually before each test. Using a multiple objective genetic algorithm, an illumination system was developed by [33] based on high power LEDs, providing sufficient radiant power and high uniformity for multi-exposure image acquisition of moving fertilizer particles. In this study, a new measurement system with four cameras was developed to accurately determine the position and velocity of fertilizer particles in three dimensions, and express these relative to a coordinate system associated with the spreader. In contrast to previous work, also the size of the individual particles could be determined. Landing positions of individual particles are calculated using a ballistic model including the effect of wind and tractor motion and from this, the spread pattern can be calculated. The system enables a fast and labor extensive determination of the spread pattern of fertilizer spreaders and will be used for calibration of fertilizer spreaders in practice. 

## 2. Materials and Methods

### 2.1. System Description

**Figure 1:**
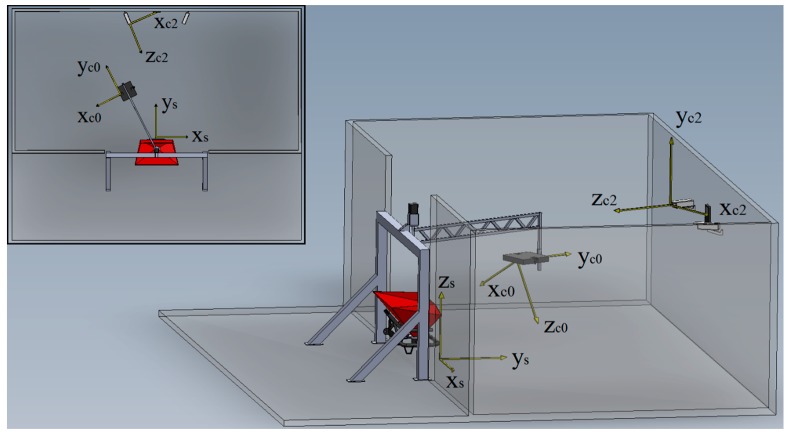
Developed setup for determining spread patterns of centrifugal fertilizer spreaders (3D view and top view). Two cameras and a LED illumination system were attached to a rotatable arm. The arm was attached to a frame under which a spreader was placed. A second set of cameras was attached to the wall of the setup. The spreader coordinate system (x_s_, y_s_, z_s_) and camera coordinate systems of camera 0 (x_c0_, y_c0_, z_c0_) and camera 2 (x_c2_, y_c2_, z_c2_) are indicated.

The setup is illustrated in Figure 1. Two monochrome cameras (Manta G-419B, Allied Vision Technologies, Stadtroda, Germany) with a resolution of 2048 × 2048 px and maximal framerate of 26 fps (at full resolution) fitted with 16 mm lenses (LM16SC, Kowa Optimed Deutschland GmbH, Duesseldorf, Germany) were used to acquire images of particles leaving the spreading vanes. The cameras, from here on called the first camera system (consisting of camera 0 and 1), were attached to the rotatable (in the horizontal plane) arm (1.8 m in length) of a custom designed stainless steel frame which was fixed to the floor. The cameras were mounted 370 mm apart, and were inclined to maximize their shared field of view (the angle between the cameras was 38.5°). The LED illumination system, developed in the study of [33], was fitted between the cameras and was used for multi-exposure image acquisition. The arm was driven by a stepper motor (Nema 42, OMC Corporation Limited, Nanjing, China), fitted to a reduction gearbox (1:100 reduction ratio, PE-W-084-100G, Servo2Go, Greenville, DE, USA). A 14 bit encoder (AS5048, AMS AG, Premstaetten, Austria) was fitted to the axle of the arm to provide the angular position. 

Two other monochrome cameras (Mako U-503B, Allied Vision Technologies), from here on called the second camera system (consisting of cameras 2 and 3), with a resolution of 2592 × 1944 px and maximal framerate (at full resolution) of 14 fps, fitted with 6 mm lenses (LM6NCM, Kowa Optimed Deutschland GmbH) were used to determine the position of the spreader and the ground plane. They were statically mounted to a stainless steel frame of 2 m high, which was fixed to the floor. The distance between the cameras was 2.35 m with an angle of 23.5° between the cameras. Around the measurement system, a wooden frame (10 m × 5 m) with black plastic cover was built to create a dark environment (exclude sunlight). To reduce bouncing back of particles after impacting the walls, strips of sackcloth were attached to horizontal bars attached to the frame. A dedicated computer (Intel Core i5-6400 CPU, 2.70 GHz, 32 Gb RAM) was used for data acquisition and processing. All software was written in C++ with the OpenCV library (Open Source Computer Vision Library, version 2.4.11). Matlab (v.2015a, Mathworks Inc., Natick, MA, USA) was used for production of the graphics in this paper.

### 2.2. Calibration

Cameras were calibrated with a custom made real-time camera calibration application, based on the OpenCV library (Open Source Computer Vision Library, version 2.4.11). For calibration, symmetrical patterns of circular markers (circles with diameter 25 mm and 120 mm with distance 35 mm and 192 mm in between for the first and second camera-system, respectively) were used. The intrinsic parameters (camera matrix and distortion coefficients) of each camera were determined. The Root Mean Square (RMS) reprojection error after calibration was generally below 0.15 px. Next, the extrinsics (position and pose) of the first and second camera system were determined. The first camera system was attached to the camera arm and moved with constant velocity during an experiment while the second camera system was statically fixed to the wall (see Figure 1). The (dynamic) transformation matrix between the first and second camera system was calculated based on the extrinsics between the first and second camera coordinate system at one position, the encoder value and the rotation vector of the arm. The latter was determined by attaching a sphere to the camera arm and determining the centroid at different arm positions in the second camera coordinate system (the details will not be discussed here).

### 2.3. Triangulation

When a point in the 3D space (in this case the centroid of a fertilizer particle) is visible in the images of two cameras with known intrinsic camera parameters and pose, the position of this point can be calculated. This process is called triangulation. In theory, the problem relies in finding the intersection point of the two rays in space. However, because of noise, the rays will not intersect. Different approaches can be used to solve this problem [34]. In this study, we used the linear triangulation method, which is often used in computer vision applications. The commonly used Linear-LS method was used to determine the 3D coordinates [34].

### 2.4. Spreader Coordinate System

The particle positions and velocities and the subsequently calculated spread pattern must be expressed in a coordinate system associated to the fertilizer spreader and the ground surface. To calibrate fertilizer spreaders in practice, it is important to determine the spread pattern of a spreader in its used configuration (as attached to the farmer’s tractor). To determine the spreader coordinate system (see Figure 1) accurately and automatically, the second camera system was used. The orientation of the ground plane was determined based on a number of images of the calibration pattern on the ground. The 3D points of the centers of the circular markers were determined and the best fitting surface was determined using linear least squares. Afterwards, the result was corrected for the height of the calibration plate above ground. The position and horizontal orientation of the spreader relative to the camera frame was determined before each spreading test by attaching two magnetic tracking circular markers (140 mm in diameter) to the back of the spreader, with equal distance to the centerline of the spreader. The center point of the markers was determined in 3D and from this, the spreader center point was determined. The horizontal spreader orientation relative to the camera system was determined based on the line through both marker center points. Based on the orientation of the camera system relative to the ground plane, the position to the spreader center and the horizontal orientation of the spreader, the spreader coordinate system was defined as the right handed Cartesian coordinate system with xy-plane coincident with the ground plane, origin going through the vertical projection of the spreader center point, x-axis oriented along the spreader frame, y-axis in the negative driving direction and the z-axis pointing upwards. The transformation matrix ($T_{CCS_{1}-SCS}$) between the first camera coordinate system and the spreader coordinate system was determined.

### 2.5. Determining Particle Parameters

#### 2.5.1. General

The next step consisted of determining the parameters of the fertilizer particles that have left the spreading vanes and expressing them relative to the spreader coordinate system. During exposure of each image, two flashes with a specific delay time were given with the illumination system. Because of this, two subsequent positions of the particles were acquired on each image. The duration of each flash determines the intensity level of the particles on the image, but also the amount of motion blur, because particles can move with velocities ranging from 10–45 m/s [32], combined with the particle velocities, determines the disparity (difference in position) between both particle positions. The delay time was calculated based on the expected particle velocity. Image processing algorithms were developed to determine the 3D position, 3D velocity and diameter of individual fertilizer particles from the images.

#### 2.5.2. Preprocessing

In a first step, a median filter was applied to reduce noise. A kernel size of 3 × 3 px was found optimal for our application (trade-off between noise reduction and image blur). A global thresholding operation was used to segment particles from background. This was possible due to the controlled environment, the uniformity of the illumination and the black background. A binary image was generated from the thresholding operation. The contours of the connected components were determined using the algorithm of Suzuki [35] for further processing. The pixels within connected components (regions) were localized for further processing. The perimeter and area of the regions were determined to calculate their convexity γ (-):

(1) $$\gamma =\frac{A}{A_{hull}}$$

where *A* is the area of the region [px]; *A_hull_* the area of the convex hull [px]; and circularity η (-):

(2) $$\eta =\;\frac{4\;\pi \;A}{P^{2}}$$

where P is the perimeter of the contour [px].

The three descriptors: area, convexity and circularity were used for particle selection. Empirically following intervals were determined: [*A_min_*, *A_max_*], [*γ_min_*, *γ_max_*] and [*η_min_*, *η_max_*]. Each region with its features falling in all three ranges was accepted as a particle.

For each particle, the image moments were determined (*m* = [0, 1], *n* = [0, 1]):

(3) $$m_{n,m}\;=\underset{x}{\sum }\underset{y}{\sum }x^{n}y^{m}I_{x,y}$$

where *I_x,y_* is the pixel value at position *x*, *y*.

From this, the centroid was calculated as follows:

(4) $$P\;=\left[\frac{m_{10}}{m_{00}},\frac{m_{01}}{m_{00}}\;\right]$$

where $m_{00}$, $m_{01}$ and $m_{10}$ are the image moments of the particle [px].

The particle centroids were undistorted by using the internal camera parameters, determined by calibration. For each particle i in the first image (camera 0), the epipolar line (the line in the second image on which the centroid of the particle geometrically should be residing) in the second image (camera 1) was calculated using the external camera parameters. Each line was encoded by three parameters (*a_i_*, *b_i_*, *c_i_*):

(5) $$a_{i}x_{j}+b_{i}y_{j}+c_{i}=0$$

#### 2.5.3. Position and Diameter

The 3D position was determined based on the position of the particle on the images of both cameras and on the intrinsic and extrinsic parameters of the camera system (see section camera calibration and triangulation).

**Figure 2:**
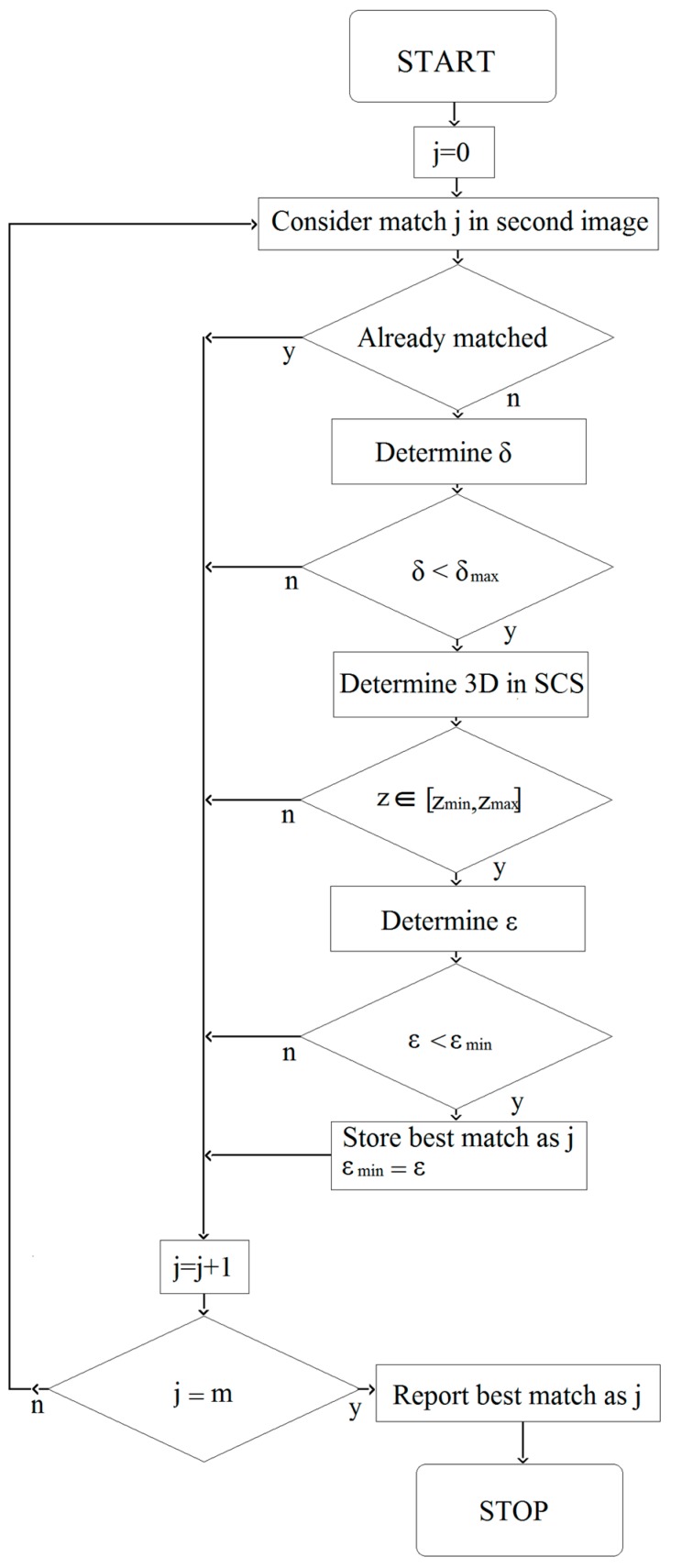
Flowchart of stereo matching algorithm. For each particle in the first camera, the match j between particles in the second camera is searched. With m the total nb of particles in the second camera image.

To triangulate the 3D position of the particles relative to the camera coordinate system of the first camera coordinate system, the position in both camera images needs to be known. The matching between the two camera images, i.e., stereo-matching was done by using the following algorithm (see Figure 2). Firstly, for each particle *i* in the first image, the corresponding particle j in the second image was searched. The distance to the epipolar line (δ) was determined as:

(6) $$\delta =\left|\frac{a_{i}x_{j}+b_{i}\;y_{j}+c_{i}}{\sqrt{{a_{i}}^{2}+{b_{i}}^{2}}}\;\right|$$

where (*a_i_*, *b_i_*, *c_i_*) are the parameters of the epipolar line of particle *i* in the second image; *x_j_*, *y_j_* describe the position of particle *j* in the second image.

In the ideal case, the distance from the particle to the epipolar line of its match would be very low (subpixel) and the matching process would be simple. However, because of the different viewpoint of the cameras and the fact that particles are not perfectly spherical or due to illumination variability, there can be a deviation between the geometric center of the projection of the particle and the projection of the true particle centroid. Therefore, all particles with an epipolar distance smaller than a pre-set value δ_max_ were considered as possible matches and their 3D position was determined in the camera coordinate system of the first camera (*C*_0_) and from this set of candidates, the true match was searched. Using the transformation matrix between the first and second camera coordinate system ($T_{C_{0}-C_{1}}$), the position of the particle in the second camera coordinate system (*C*_1_) was determined.

The position in the spreader coordinate system (***P>**_S_*) was determined based on the transformation matrix between the first camera coordinate system and the spreader coordinate system $T_{C_{0}-S}$:

(7) $$P_{S}=T_{C_{0}-S}\;P_{C_{0}}\;$$

Information on the spreading process was used as well. Fertilizer particles thrown by a spreader move at a certain height above the ground. When the z-coordinate of the particle in the spreader coordinate system was part of the interval [*h_min_*, *h_max_*], the match was approved and the stereomatching coefficient was calculated:

(8) $$ℇ=\;k_{1}\frac{\left|P_{S,z}-h_{mean}\right|}{h_{mean}}+k_{2}\;\frac{\left|\eta_{i}-\eta_{j}\right|}{\frac{\eta_{i}+\eta_{j}}{2}}+k_{3}\;\frac{\left|D_{i}-D_{j}\right|}{\frac{D_{i}+D_{j}}{2}}$$

where *k*_1_, *k*_2_ and *k*_3_ weighting coefficients, determined empirically [-] when *D*_i_ is the diameter of the particle based on the particle area in the first image (*A_i_*):

(9) $$D_{i}=\;2\sqrt{\frac{A_{i}}{\pi }}\frac{P_{C_{1},z}}{f_{1}}\;$$

and *D*_j_ the diameter of the particle based on the area of the particle’s match in the second image (*A_j_*)

(10) $$D_{j}=\;2\sqrt{\frac{A_{j}}{\pi }}\frac{P_{C_{2},z}}{f_{2}}\;$$

The match with the smallest value for ℇ was considered as the best match. The 3D position in the spreader coordinate system (*P_S_*) was approved. The particle diameter was calculated as the average value between *D_i_* and *D_j_*. 

To verify the calculation of the particle diameter using image processing, an experiment with plastic spheres with known diameter (5.96 mm ± 0.005) was conducted. The spheres were launched one by one (50 in total) with an average velocity of approximately 25 m/s using an air pressure device which was mounted horizontally at 90 cm above ground. The average absolute difference between the true diameter and the measured diameter was calculated. 

#### 2.5.4. Velocity

To determine the velocity of the particles, the two subsequent positions on the multi-exposure (two flashes) images must be found. In the previous step, the 3D position of each particle position was found. After matching the two positions for each particle, i.e., time-matching, the velocity in 3D can be determined. Compared to the stereo-matching process, the number of possible matches is significantly higher. Particles were not matched using traditional feature descriptors and matchers because particles can geometrically be very similar and only two-dimensional information is used. Furthermore, due to particle spin, their two-dimensional projection (associated with each flash) can change. The time-matching algorithm presented here is based on four factors. Three are image related: the particle shape, area and the direction of motion. The fourth matching factor is the position relative to the spreader coordinate system.

For each particle, all candidate matches are determined. A particle was considered a candidate match when the disparity (difference in 2D position) and direction of motion fall within a certain range and when the difference in height above ground between the two positions does not exceed a maximal value. For each match, a matching coefficient (ζ) was calculated as follows:

(11) $$\zeta =\;k_{4}\left(1-\frac{\left|A_{p}-A_{q}\right|}{\frac{A_{p}+A_{q}}{2}}\right)+k_{5}\left(1-\;\frac{\left|\eta_{p}-\eta_{q}\right|}{\frac{\eta_{p}+\eta_{q}}{2}}\right)$$

where *A_p_*, *A_q_*, η*_p_*, η*_q_* represent the particle area (px) and circularity (-) of two particles *p* and *q*; *k*_4_, *k*_5_ weighting coefficients, determined empirically (-).

After determining similarity coefficients for all particles, the matches were analyzed. For each particle that was not matched already, the number of possible candidates was checked. When there was only one candidate, the matches of this candidate were checked. If the particle was the best match (largest matching coefficient) of the candidate, the match was approved and both particles were removed from the stack of particles that still needed to be matched. In case there were multiple candidates, and the candidate had the particle as best match, the match was added to a vector of possible matches. The vector of possible matches was then checked and from this vector, the candidate with the highest matching coefficient was approved as final match. After matching, the particle diameter was calculated as the average diameter between the two positions.

The 3D velocity ($v_{p,SCS})$ was determined based on the 3D position at both particle instances (*t* and *t* + Δt) and the time between the two subsequent flashes of the stroboscope (Δt):

(12) $$v_{p,S}=\;\frac{P_{S}\left(t+\Delta t\right)-P_{S}\left(t\right)}{\Delta t}$$

where $P_{s}$ is the 3D position of the particle instances (m) in the spreader coordinate system Δt the time between two subsequent flashes (s).

The particle positions and velocity vectors are expressed relative to the spreader coordinate system, associated with the fertilizer spreader, which is static during the test, but moving while spreading in the field. Therefore, vectors in the spreader coordinate system need to be transformed to a static world coordinate system (*W*) to account for the tractor driving speed (***v****_t_*):

(13) $$v_{p,W}=v_{p,S}+v_{t,S}$$

### 2.6. Spread Pattern

To determine the landing position of a fertilizer particle during spreading, the wind speed (***v****_w_*) needs to be taken into account. The velocity vector of the particle relative to the air ***v****_p,rel_* was calculated as follows:

(14) $$v_{p,rel}=v_{p,W}-\;v_{w,\;W}$$

During their motion in the air, particles are subjected to gravitational and drag forces. The drag coefficient $C_{d}$ depends on fluid and particle properties, which are expressed in the dimensionless Reynolds number (*Re*):

(15) $$Re=\;\frac{D\;\left|{\vec{v}}_{rel}\right|\;\rho_{air}}{\mu }$$

where *D* is the particle diameter (m), *µ_air_* the dynamic viscosity (kg m^−1^ s^−1^) and $\rho_{air}$ the density of air (kg/m³).

Similarly as in [8], the drag coefficient (*C_d_*) was calculated as follows:

(16) $$C_{d}=\frac{30}{Re}+67.289e^{-5.03\phi }$$

where ϕ is the sphericity of the particle (-).

The particle mass *M* (kg) was calculated as:

(17) $$M=\rho_{p}V$$

with:

(18) $$M=\frac{4}{3}\pi {\left(\frac{D}{2}\right)}^{3}$$

and ρ*_p_* the particle true density (kg/m^3^).

The following system of differential equations describes the 3D trajectory of particles with respect to the spreader coordinate system:

(19) $$\left\{ \begin{array}{c} \frac{d^{2}x}{dt^{2}}=-\;C_{d}\frac{A_{proj}\;\rho_{air}}{2M}\left|{\vec{v}}_{rel}\right|v_{rel_{x}}\\ \frac{d^{2}y}{dt^{2}}=-\;C_{d}\frac{A_{proj}\;\rho_{air}}{2M}\left|{\vec{v}}_{rel}\right|v_{rel_{y}}\\ \frac{d^{2}z}{dt^{2}}\;=\;-C_{d}\frac{A_{proj}\;\rho_{air}}{2M}\left|{\vec{v}}_{rel}\right|v_{rel_{z}}-g\\ C_{d}=f\left(Re,\phi \right)\\ Re=f\left(\left|{\vec{v}}_{rel}\right|\right) \end{array}\right.$$

where *g* is the gravitational acceleration (m s^−1^).

The differential equations, given in Equation (6) are non-linear and of second order and were therefore solved numerically. Similarly to [8], Euler’s forward solution was used, providing a good accuracy at small enough time steps [36].

The landing positions were transformed to a grid (51 × 35, resolution 1 m^2^). Each cell of the grid at position (*x* = *i*, *y* = *j*) contains the mass of all particles landing in that cell (*S_i,j_*). The Pearson coefficient of correlation was used to determine the similarity between spread patterns (*α*, *β*) in two spatial dimensions:

(20) $$\rho_{\alpha ,\beta }=\frac{Cov_{\alpha ,\beta \;}}{\sigma_{\alpha }\sigma_{\beta }}$$

where σ represents the standard deviation:

(21) $$\sigma =\sqrt{\frac{1}{n-1}\;\overset{n_{x}}{\underset{i=1}{\sum }}\overset{n_{y}}{\underset{j=1}{\sum }}{\left(S_{i,j}-\bar{S}\right)}^{2}}$$

where $\bar{S}$ is the the average mass per grid cell (kg) and ${\mathrm{Cov}}_{\alpha ,\beta }$ the covariance between the two spread patterns:

(22) $${\mathrm{Cov}}_{\alpha ,\beta }=\frac{1}{n-1}\;\overset{n_{x}}{\underset{i=1}{\sum }}\overset{n_{y}}{\underset{j=1}{\sum }}\left(S_{\alpha_{i,j}\;}-{\bar{S}}_{\alpha }\right)\left(S_{\beta_{i,j}}-{\bar{S}}_{\beta }\right)$$

To enable comparison with spread patterns obtained in the field, which are traditionally measured perpendicular to the driving direction, results were transformed to one dimension. This was done by summing the mass of all the cells in the driving direction. For clarity, only the spread pattern of the left disc was measured. This was done by closing the orifice feeding the right spreading vane. In practice, however, both discs are used. Similar to [12], the spread pattern of the right disk was obtained by mirroring the transverse spread pattern of the left disk. Because the spreader coordinate system was situated at the spreader center, this was done relative to position *x* = 0. The spread pattern of both discs was then obtained by simple superposition of the left and right spreading patterns assuming no interaction, e.g., collision, between particles from the different disks. Because in the field, the tractor is moving, the resulting values were rescaled to calculate the actual dosage applied per unit area. For centrifugal spreaders, subsequent spread patterns need to overlap to ensure a homogeneous distribution. The total transverse spread pattern, i.e., the transverse spread pattern of the spreader taking into account multiple swaths was calculated based on overlapping the spread pattern of three subsequent swaths [37] at the working width specified by the manufacturer. The coefficient of variation (CV, %) was calculated based on the data between the centre point of the first swath to the centre point of the third swath:

(23) $$CV=\frac{\sigma_{t}}{\mu_{t}}100$$

where $\mu_{t}$ and $\sigma_{t}$ are the mean and standard deviation (kg/ha).

The min-to-max ratio (-) was defined as:

(24) $$\frac{\min\left(S_{t,i}\right)}{\max\left(S_{t,i}\right)}$$

where *S_t,i_* is the mass of fertilizer at *x* = *i*.

### 2.7. Experiments

Spreading experiments were conducted with a commercially available fertilizer spreader. The physical properties of the fertilizer used during the experiments were determined in [8] and are given in Table 1. The particle size distribution measured by sieving (according to standard [38]) was used to verify the values measured by the system developed in this research.

The spreader used in the experiments was a new Vicon RO-XL (VN236, Kverneland Group, Klepp Stasjon, Norway), equipped with disks containing six longer vanes (285 mm long, 60 mm high) and two shorter vanes (80 mm long, 60 mm high) at the standard configuration provided by the manufacturer. The vanes were straight and mounted on a flat disk. The spreader was set according to the manufacturer setting tables. Two different configurations were used, as illustrated in Table 2.

For each experiment, two scans were performed, meaning that the rotating arm moved twice in a continuous way from its begin to end position during one experiment. Each scan took 60 s, and 1500 images were taken (25 fps). Two replicates were performed for each spreader configuration. The spreader was filled with 300 kg fertilizer before each test (six separate bags of 50 kg). The parameters used for the image processing algorithms are given in Table 3.

Field experiments were conducted to compare the obtained transverse spread pattern at similar spreader configurations. A total of 100 collection trays (0.5 m × 0.5 m) were placed on one row of ladders, perpendicular to the driving direction of the tractor. Trays were removed at the positions of the tractor wheels. Results were transformed to a resolution of 1 m × 1 m to compare with the transverse spread patterns obtained with the camera system. During the field experiments, the average wind velocity and direction were measured using a local weather station at 5 min intervals (05103-L sensor on a CR1000 datalogger, Campbell Scientific, Logan, UT, USA). The tractor driving direction was determined by measuring two points of the trajectory with a GNSS receiver (S10, Stonex, Lissone, Italy) using the Flemish Positioning Service (FLEPOS) correction signals. Based on this, the wind velocity vector was calculated in the spreader coordinate system (see Table 4) and used for calculating the spread pattern with the camera system.

## 3. Results and Discussion

**Figure 3:**
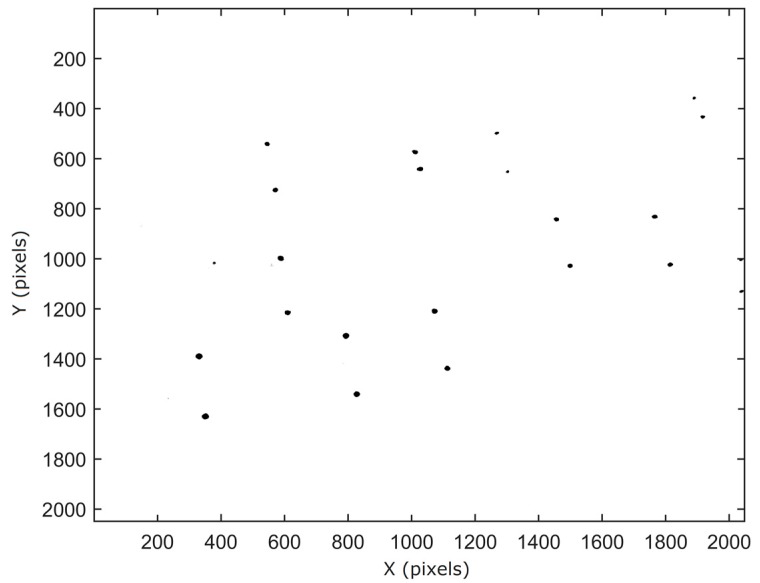
Multi-exposure image taken from particles moving underneath the first camera system (inverted image after thresholding operation; units in image coordinates). The spreader was set at configuration A.

Figure 3 illustrates a multi-exposure image, taken from fertilizer particles moving underneath the first camera system (inverted view). The disparity between the subsequent particle positions depends both on the distance to the camera and on the velocity of the particles.

Table 5 gives the median distance to the epipolar line for particles from both replicates for spreader configuration B. The 10th and 90th percentile are given as well. Generally, it can be seen that the median distance lies below 0.47 px and that the value was slightly smaller for configuration B. This indicates that the stereo-matching algorithm performed well. The 10th and 90th percentile illustrate the differences between different particles. This can have multiple causes. Particles in the first camera image can be matched incorrectly to other particles with similar shape and size that are coincidentally on the epipolar line of the other camera image. However due to feedback on the calculated 3D position, this is unlikely to happen. Another cause can be the non-sphericity of the particles and the fact that the cameras use two different viewpoints and therefore, the particle centers on both images do not represent exactly the same 3D point. It was visually checked that in most cases, the algorithm resulted in a correct match.

**Figure 4:**
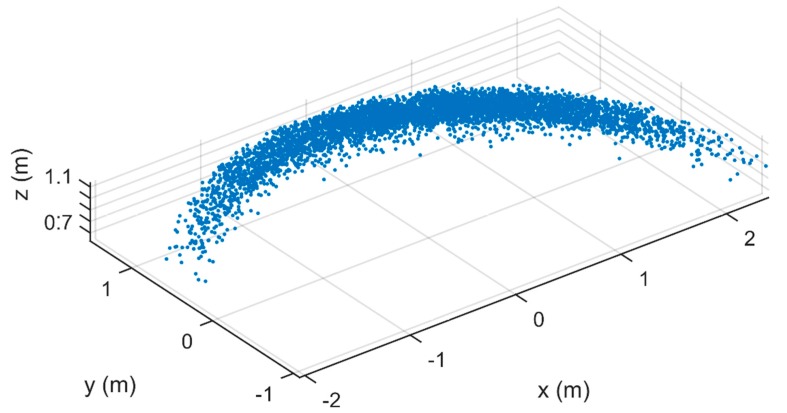
Particle 3D positions in spreader coordinate system. The spreader was set at configuration B.

Figure 4 illustrates the positions of the individual particles in the air, expressed in the spreader coordinate system. In this case, the positions of 5270 particles were determined. The particle positions are situated around a circle segment, because they are acquired by cameras that are mounted on a rotating arm and particles are only registered when they pass below this camera setup. The figure does not represent a single throw, but a collection of particles that move were captured on image.

**Figure 5:**
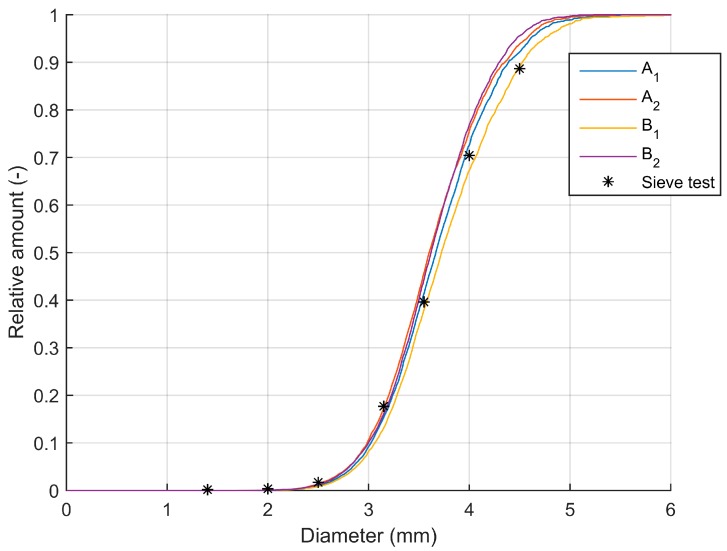
Cumulative particle size distribution (mass based). Results from image processing are given (two tests of configuration A and B), as well as the results of the sieve test.

To verify the calculation of the particle diameter, an experiment with moving plastic spheres with known diameter was conducted. The average absolute difference between the true diameter and the measured diameter was 0.0671 mm (σ = 0.0422 mm) indicating a very high accuracy of the system. From Table 6 and Figure 5, it can be seen that the size distribution of the fertilizer particles is similar between the replicates of each spreading test. 

The size distribution for the first test at configuration B is slightly larger compared to the second test. This can be caused by the fact that, although the same fertilizer type (from the same manufacturer) was used between tests, small differences between different batches can be found (measurements were performed using multiple separate bags of 50 kg). The distribution is also comparable between the two spreader configurations even though at configuration B, a higher disk rotational velocity was used. This suggests that there is no increase in the breaking of particles due to a larger impact with the vanes. This can be attributed to the design of the spreader, which uses a centered feeding in contrast to most other spreader manufacturers on the market. Figure 5 also shows that the particle size distribution is comparable to the distribution measured with the sieve test. In three of the four tests, a slightly smaller amount of large particles (>4.5 mm) was found compared to the sieve test. An increase in particle strength with an increase in diameter for CAN fertilizers was found by [39], indicating that this finding does not relate to a higher breaking percentage of larger particles. As mentioned before, small differences between different batches of the same fertilizer can exist which could be the reason for this small difference. Furthermore, during sieving, particles fall through the sieve if their size in either orientation is smaller than the mesh size. It is therefore possible that the sieve test underestimates the true particle. In case particle spin occurs [22], the camera system determines the diameter of each particle based on four different projections (two positions for each camera) and can therefore be more accurate.

**Figure 6:**
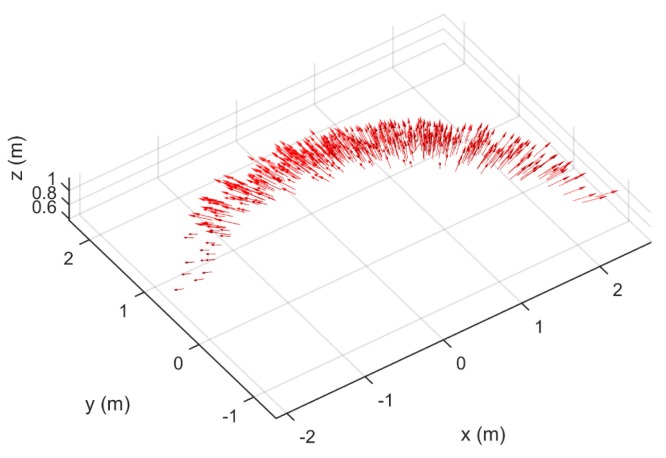
Velocity vector plot of particles expressed in spreader coordinate system. The spreader was set at configuration B. For reasons of clarity, only 10% of the particles are shown.

**Figure 7:**
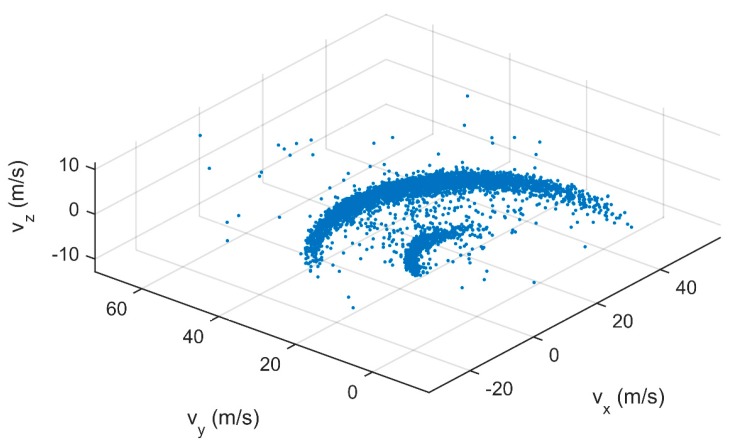
Particle 3D velocity components expressed in spreader coordinate system. The spreader was set at configuration B.

**Figure 8:**
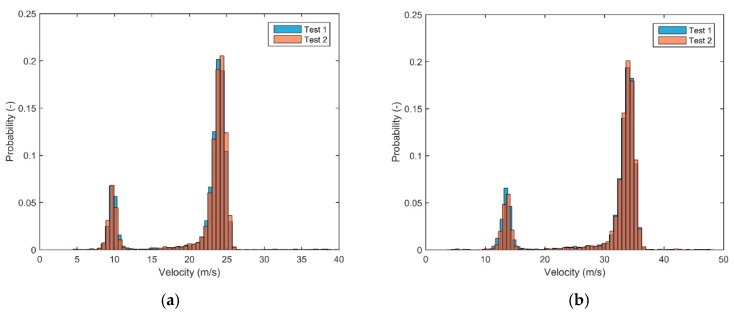
Histograms of resulting velocities for configuration A (**a**) and B (**b**). The overlap is given in brown.

The velocity of the particles was determined based on two subsequent 3D positions using the multi-exposure technique. For each particle, a 3D velocity vector was determined based on the time matching algorithm explained in the materials and methods section. Figure 6 shows the velocity vector for a small amount of particles (10% of the total). Figure 7 represents the 3D velocity components for all the particles at configuration B (first replicate). It should be noted that the points thus do not represent particle positions. Two groups of velocities can be identified. These represent particles that are thrown with the small and large vanes of the spreader respectively. The groups appear to be situated around circle segments, indicating a similar resulting velocity. It can be seen that some particles have slightly different velocity components, this can be caused by impact of particles with e.g., the camera frame, by rebounding particles or by a wrong match by the time-matching algorithm. Particles are not perfectly spherical and can spin during the ballistic flight [22]. Therefore, the shape of the 2D projection between the two subsequent positions can change, which can have an effect on the matching process. This is why, in contrast to [32], no fixed window-based correlation methods were used for the matching process. Generally, it can be seen that the number of particles with abnormal velocity components is very limited indicating a good performance in the prediction of the velocity vectors. In Figure 8, histograms of the resulting velocity are shown for both replicates of configuration A and B. Each histogram shows two peaks, related to the two groups of velocities that were found on Figure 7 (the resulting velocity is the magnitude of the vector). The reason for these two peaks is that two types of vanes were used on the spreading disk: six long ones and two short ones. The two short vanes are responsible for the small peak in the histograms (at lower velocities) while the six longer vanes are responsible for the large peak in the histograms (at higher velocities). Histograms for both replicates are very similar, indicating a high repeatability of the measurements. Minor differences can be caused by the spreader, e.g., small changes in the mass flow rate.

**Figure 9:**
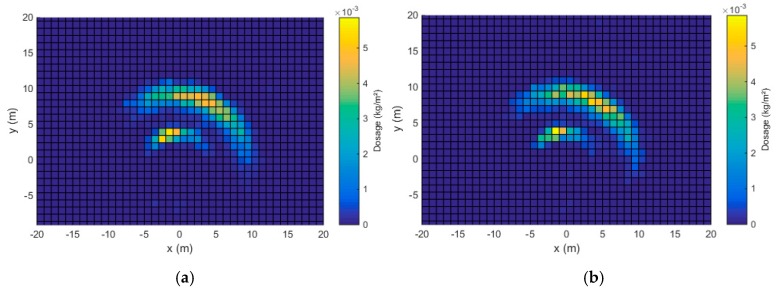
Static spread pattern, configuration A, test 1 (**a**) and test (**b**).

**Figure 10:**
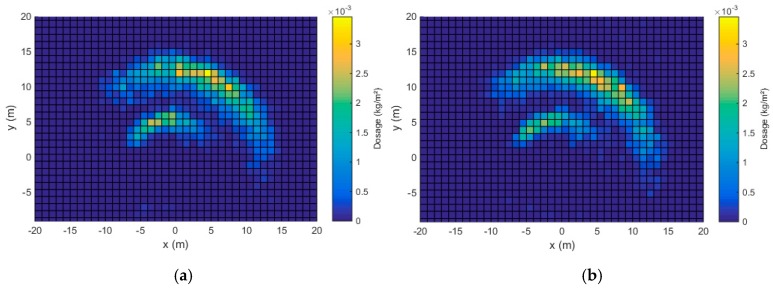
Static spread pattern, configuration B, test 1 (**a**) and test 2 (**b**).

The static spread patterns for both tests for both spreader configurations are given in Figure 9 and Figure 10. As mentioned before, only the left spreading disk was used during the experiments. Therefore, the resulting spread pattern was asymmetric around the centre-point. The area covered by the spread pattern clearly increased because of the larger disk rotational speed, which is necessary to obtain a larger working width. Each spread pattern has two c-shaped parts, the smallest part is caused by slower moving particles, ejected by the two smaller vanes. The larger part is caused by particles that are ejected by the longer vanes, giving the particles more radial and tangential velocity [16]. The correlation coefficients between the two replicates for both configurations are given in Table 7. The results indicate a high repeatability. The correlation coefficient is slightly smaller for configuration B, which can be attributed to the larger spreading area and increased sensitivity for errors.

**Figure 11:**
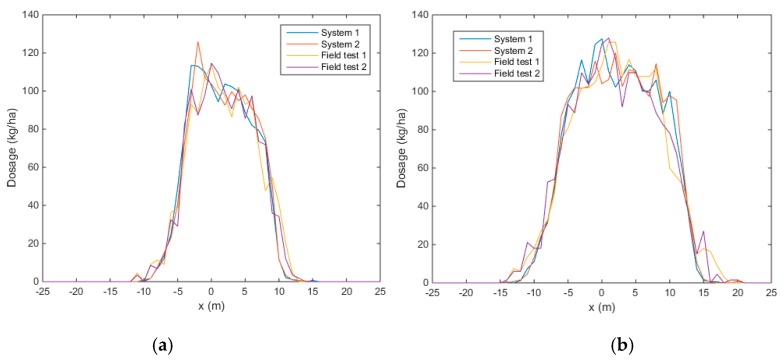
Transverse spread pattern, configuration A (**a**) and B (**b**). Two replicates were measured with the camera system (system) and in the field using collection trays (field).

The transverse spread patterns measured with the system and in the field are given in Figure 11. Table 8 gives the correlation coefficients for the transverse spread patterns. It can be seen that the correlation coefficient for the two replicates with the newly developed measurement system is larger compared to the field measurements, for both spreader configurations. The repeatability for the camera system is thus higher compared to the repeatability of the field measurements. The variability between the replicates of the field measurements are in line with results reported by [12]. This was expected because more external factors can influence the spreading process. The unevenness of the terrain can have an influence on the mass flow rate at a certain moment, but also on the parameters of the particles leaving the spreading disks. Although the wind velocity and direction were measured, short term fluctuations can occur which affect the trajectory of particles in the air [8]. Because of the grass in the field, particles are expected to bounce less compared to measurements on a solid surface such as concrete. Despite the anti-reflection grids in the collection trays, particles can bounce out of the collection trays [12,40] and, dependent on the collection method, different results can be obtained [41].

**Figure 12:**
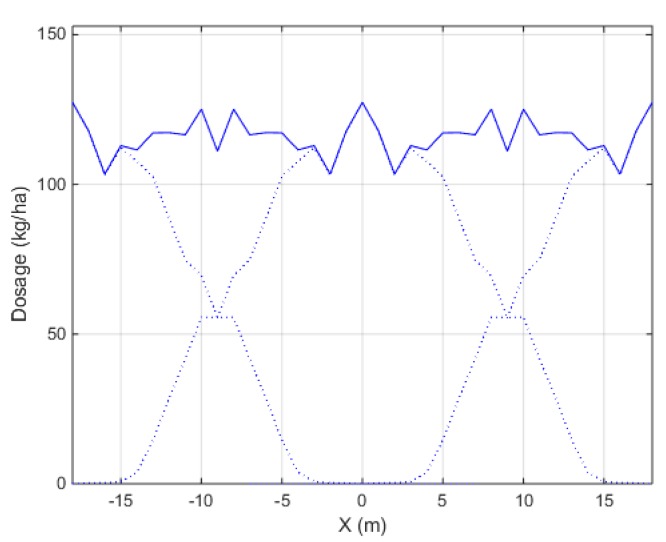
Total transverse spread pattern (solid line) with overlap between spread patterns of subsequent swaths (dashed line). The spread pattern was measured by the camera system at configuration B (18 m working width, first replicate). A CV value of 5.66% was found.

Table 9 gives the min-to-max ratio and Coefficient of Variation for the total transverse spread pattern after overlapping subsequent swaths. Results are given for both spreader configurations, measured with the camera system and with the collection trays on the field. Figure 12 illustrates the total transverse spread pattern for one measurement with the camera system at spreader configuration B. From Table 9, it can be concluded that the field tests showed a slightly larger difference in min-to-max ratio and CV between replicates compared to the camera system. This was expected because the correlation between spread patterns was larger for the system (see Table 8). Generally, the spreader performs well, since all CV values are far below 15% which is considered as the acceptable limit for centrifugal spreaders in practice [9,42]. It can be seen that between replicates with the camera system, although the static and transverse spread patterns show high similarity (see Table 8), the calculated CV can still vary slightly, 2.36% and 1.76% for configuration A and B respectively. This indicates a high sensitivity for the CV. This can be caused by the fact that overlap of subsequent swaths is necessary to achieve a uniform distribution on the field. Therefore, differences between replicates in the overlap zone of the transverse spread pattern have a larger effect compared to the center of the spread pattern.

The system developed in this study can be used for spreader adjustment in practice. The spread pattern can be determined fast and accurately without depending on the spreader configuration, meaning that the same methodology can be applied for each broadcast spreader type and configuration which was not the case in the study of [7]. The spreader can thus be measured in the configuration (spreader settings, mounting height and orientation) that is used by the farmer. In case the results are unsatisfactory, spreader parameters can be adjusted to correct the spreading process to ensure a homogeneous distribution in the field. The spread pattern of unknown and new fertilizers can be determined fast and accurately. For example, in case of spreading organomineral fertilizers, which are suitable for broadcast spreading [43] but for which no setting tables exist. The system can be of interest for spreader manufacturers as a tool to identify particle parameters after ejection. It gives a lot more insight in the spreading process than traditional collection techniques measuring the distribution of fertilizer on the ground. The prototype developed in this study requires much less space compared to traditional testing facilities and the innovative test bench (40 m × 10 m) by [31]. This means reduced costs for building test facilities and reduced running costs (heating, cleaning, air conditioning). By using this hybrid measurement approach, it is possible to include the effect of tractor motion in the calculations, which will have an effect because it reduces the velocity of the particles in the driving direction. This would be difficult to investigate with traditional collection techniques that determine the fertilizer distribution in two directions, because in that case, the spread pattern of a static spreader is determined. Wind can have a large effect on the landing positions of individual fertilizer particles [8]. Results of field tests by [44] confirm the large effect of crosswind on the transverse spread pattern. Through simulations with the system developed in this paper, it is possible to determine the effect of different wind directions and wind velocities on the spread pattern. The effect of wind and tractor velocity on the distribution of fertilizer will be investigated in future research. Finally, it would be possible to incorporate field models to calculate landing position on uneven or sloped fields. The effect will be calculated in relation to how the spreader is situated with respect to both the ground and the camera system. However, in extreme cases, this will require hardware adaptations, because the particles will be out of focus for the camera system at certain arm positions. This could be adjusted by moving the cameras and illumination system in a controlled way upwards or downwards during rotation of the camera-arm.

## 4. Conclusions

Because of the large environmental impact of fertilizer application, it is important that it be correctly distributed on the field. For granular fertilizer, mostly centrifugal fertilizer spreaders are used. Although simple in working principle, their spread pattern is prone to errors, which are often caused by lack of calibration for the specific fertilizer used. At farm level, spread patterns are traditionally measured using collection trays, which is a laborious and time consuming work. Therefore, we developed an innovative multi-camera system to predict the spread pattern in a fast and accurate way, independent of the spreader configuration. Particle position and velocity were determined in three dimensions relative to a spreader coordinate system which was determined using image processing allowing to determine particle diameters as well. Based on this, individual landing positions of particles were simulated by using a ballistic model. The effect of wind and tractor motion were also included in the calculations. Experiments were conducted with a commercially available spreader in combination with a commonly used fertilizer type. Two configurations, each with two tests, were evaluated, corresponding to a lower and higher disk rotational velocity. The system showed a high repeatability in determination of the particle size distribution, as the results were similar between tests. Furthermore, an experiment with ideal spheres showed a very accurate calculation of the particle diameter. This was confirmed by the fact that the calculated size distributions for fertilizer particles were comparable to sieve test results. The predicted particle velocities and resulting spread patterns were highly repeatable between replicates. Results were transformed to one dimensional space and compared to transverse spread patterns obtained by field experiments. The spread patterns showed a good correlation, although small differences were found between the calculated Coefficient of Variation (CV) after overlapping spread patterns for subsequent swaths. This illustrates the sensitivity of the CV for the shape of the spread pattern. The system developed in this study can be used for spreader adjustment in practice, to calibrate spreaders for new particle types, but also to gain more insight in the spreading process or as a modeling tool. In future research, the system will be used to simulate the effect of wind on the spread pattern of fertilizers in practice.

## Figures and Tables

**Table 1:** List of fertilizer physical properties for CAN fertilizer * (ammonium nitrate 27% N + 4% MgO, Scoriethom) used in this experiment. True density was determined using gas pycnometry. For density, two replicates were used. Both the bulk and true density were determined.

Parameter	Specification	Value
Density (kg m^−3^)	Bulk	1035, 1033
	True	1780, 1790
Sieve fraction ** (%)	0–1.4 mm	0.16
	1.4–2 mm	0.21
	2–2.5 mm	1.33
	2.5–3.15 mm	15.98
	3.15–3.55 mm	21.96
	3.55–4 mm	30.80
	4–4.5 mm	18.23
	>4.5 mm	11.33
Median diameter (mm) ***		3.70
Sphericity (-)		0.9655

* Supplier: Aveve Dirk Notebaert, Scheldewindeke, Oost-Vlaanderen, Belgium; ** Mass fraction; *** Determined based on linear interpolation of sieve test results.

**Table 2:** List of settings used in this study for the Vicon RO-XL (VN236) spreader. In all cases, the spreader was placed horizontally with disks 750 mm above ground. The position of the orifice was set at N and a dosage of 116 kg/ha was used.

Configuration	Working Width (m)	Disk Rotational Velocity (rpm)	Dosage Setting (-)
A	12	540	36 + 2
B	18	750	42 + 2

**Table 3:** Image processing algorithm parameters used during the experiments.

Parameter	Unit	Value
[*A_min_*, *A_max_*]	px	[50, 1200]
[*h_min_*, *h_max_*]	m	[0.6, 1.2]
[*k*_1_, *k*_2_, *k*_3_, *k*_4_, *k*_5_]	-	[0.33, 0.33, 0.33, 0.5, 0.5]
*δ_max_*	px	2
[*γ_min_*, *γ_max_*]	-	[0.91, 1]
[*η_min_*, *η_max_*]	-	[0.55, 1]

**Table 4:** Wind velocity expressed in the spreader coordinate system (***v****_w_*_,*x*_, ***v****_w_*_,*y*_). The resulting wind velocity is given as well (***v****_w,tot_*).

Configuration	Wind Velocity (m/s)		
	*v_w,x_*	*v_w,y_*	*v_w,tot_*
A	−1.93	−1.00	2.17
B	−2.10	−1.40	2.52

**Table 5:** Median distance of particle centroid to epipolar line in px (between brackets, the 10th and 90th percentile are given).

Configuration	Test 1	Test 2
A	0.4645 (0.0856, 1.2269)	0.3998 (0.0719, 1.1329)
B	0.3391 (0.0617, 0.9821)	0.3292 (0.0608, 0.9539)

**Table 6:** Particle size distribution for two tests at each spreader configuration (mass based). The 10th, 50th and 90th percentile are given (all in mm).

Configuration	Test	D10	D50	D90
A	1	3.03	3.67	4.39
	2	2.99	3.61	4.36
B	1	3.07	3.73	4.53
	2	3.00	3.63	4.30

**Table 7:** Correlation coefficients between two replicates of the static spread pattern as calculated by Equation (20). A grid resolution of 1 × 1 m was used (51 × 35 cells)

Configuration	Correlation Coefficient (-)
A	0.9803
B	0.9598

**Table 8:** Correlation coefficients between transverse spread patterns for configuration A and B measured with the camera system and with collection trays on the field. A grid resolution of 1 m × 1 m was used (51 cells).

Configuration	Comparison	Correlation Coefficient (-)
A	System 1–system 2	0.9944
	Field 1–field 2	0.9893
	System 1–field 1	0.9759
	System 1–field 2	0.9829
	System 2–field 1	0.9754
	System 2–field 2	0.9795
B	System 1–system 2	0.9920
	Field 1–field 2	0.9861
	System 1–field 1	0.9790
	System 1–field 2	0.9851
	System 2–field 1	0.9768
	System 2–field 2	0.9777

**Table 9:** Minimum to maximum ratio and Coefficient of Variation (CV) for the total transverse spread pattern at working width specified by the manufacturer (12 m for configuration A, 18 m for configuration B). Transverse distributions for both replicates with the collection trays on the field and with the system developed in this paper were used.

Spreader Configuration	Measurement System	Test Number	Min-to-Max Ratio (-)	CV (%)
A	Field	1	0.8017	5.86
		2	0.7647	8.82
	System	1	0.7917	9.42
		2	0.8366	7.06
B	Field	1	0.9141	3.20
		2	0.8072	5.56
	System	1	0.8118	5.66
		2	0.8012	7.42

## Nomenclature

**Symbol or Abbreviation**	**Unit**	**Explanation**
a, b, c	-	Line parameters in an image
A	pixels	Particle area
CAN	-	Calcium Ammonium-Nitrate fertilizer
C_d_	-	Drag coefficient
CEMIB	-	CEmagref Mineral Bench
Cov		Covariance
CV	%	Coefficient of variation
D	m	Particle diameter
f	pixels	Lens focal length
g	m s^−2^	Gravitational acceleration
h	m	Height of particle above ground
k	-	Weighting coefficient for matching
I	-	pixel intensity value
m	pixels	Image moment
M	kg	Particle mass
NUE	-	Nitrogen Use Efficiency
p	pixels	Particle perimeter
P	m	Particle position
px	-	Pixels
Re	-	Reynolds number
s	-	Scale factor
SCS	-	Spreader coordinate system
T	-	Transformation matrix (rotation and translation)
u	pixels	Image coordinates in camera image 1
v	pixels	Image coordinates in camera image 2
V	m³	Particle volume
v_p_	m s^−1^	Particle velocity
v_t_	m s^−1^	Tractor driving speed
WCS	-	World coordinate system
α, β	-	Two spread patterns
γ	-	Convexity
δ	pixels	Distance to epipolar line
Δt	s	Time between subsequent flashes
ℇ	-	Stereomatching coefficient
ζ	-	Time-matching coefficient
η	-	Circularity
μ	kg m^−1^ s^−1^	Air dynamic viscosity
ρ	-	Pearson coefficient of correlation
$\rho_{air}$	kg m^−3^	Air density
$\rho_{p}$	kg m^−3^	Particle true density
σ		Standard deviation
ϕ	-	Sphericity
